# Nitric oxide production and antioxidant function during viral infection of the coccolithophore *Emiliania huxleyi*

**DOI:** 10.1038/s41396-018-0325-4

**Published:** 2019-01-03

**Authors:** Brittany M. Schieler, Megha V. Soni, Christopher M. Brown, Marco J. L. Coolen, Helen Fredricks, Benjamin A. S. Van Mooy, Donald J. Hirsh, Kay D. Bidle

**Affiliations:** 1 Department of Marine and Coastal Sciences, Rutgers University, 71 Dudley Road, New Brunswick, NJ 08901 USA; 2 WA-Organic and Isotope Geochemistry Center, School of Earth and Planetary Sciences, Curtin University, Bentley, WA 6102 Australia; 3 Department of Marine Chemistry and Geochemistry, Woods Hole Oceanographic Institution, Woods Hole, MA 02543 USA; 4 Department of Chemistry, The College of New Jersey, Ewing, NJ 08628 USA

**Keywords:** Cellular microbiology, Water microbiology

## Abstract

*Emiliania huxleyi* is a globally important marine phytoplankton that is routinely infected by viruses. Understanding the controls on the growth and demise of *E. huxleyi* blooms is essential for predicting the biogeochemical fate of their organic carbon and nutrients. In this study, we show that the production of nitric oxide (NO), a gaseous, membrane-permeable free radical, is a hallmark of early-stage lytic infection in *E. huxleyi* by Coccolithoviruses, both in culture and in natural populations in the North Atlantic. Enhanced NO production was detected both intra- and extra-cellularly in laboratory cultures, and treatment of cells with an NO scavenger significantly reduced viral production. Pre-treatment of exponentially growing *E. huxleyi* cultures with the NO donor S-nitroso-N-acetylpenicillamine (SNAP) prior to challenge with hydrogen peroxide (H_2_O_2_) led to greater cell survival, suggesting that NO may have a cellular antioxidant function. Indeed, cell lysates generated from cultures treated with SNAP and undergoing infection displayed enhanced ability to detoxify H_2_O_2_. Lastly, we show that fluorescent indicators of cellular ROS, NO, and death, in combination with classic DNA- and lipid-based biomarkers of infection, can function as real-time diagnostic tools to identify and contextualize viral infection in natural *E. huxleyi* blooms.

## Introduction

*Emiliania huxleyi* is a cosmopolitan species of coccolithophore, a group of unicellular, eukaryotic marine phytoplankton that produces intricate shells (coccoliths) of calcium carbonate. As both a dominant calcifier and a bloom-forming photoautotroph, *E. huxleyi* exerts a profound influence on marine biogeochemical cycles (particularly of carbon and sulfur) and food web dynamics. *E. huxleyi* is known for forming large annual blooms in the North Atlantic, often spanning >10^5^ km^2^ with cell densities exceeding 10^6^ cells l^−1^ [1–3]. These blooms are associated with dimethyl sulfide production in the surface ocean and flux into the atmosphere [4], increased albedo and heating of the surface ocean due to their coccoliths [5], and enhanced export flux of carbonate to the deep ocean [6].

Understanding the mechanisms of *E. huxleyi* bloom termination is necessary for predicting the fate of its calcium carbonate and fixed organic carbon in the ocean. *E. huxleyi* is often infected by large, double stranded DNA viruses belonging to the group *Phycodnaviridae* [7–9]. These Coccolithoviruses, known as EhVs, have been shown to cause the termination of *E. huxleyi* in culture [10, 11] and natural blooms [12–16]. One paradigm holds that viral lysis shunts fixed carbon away from downward vertical flux or transfer to higher trophic levels by stimulating cell lysis and bacterial respiration of dissolved organic carbon in the surface ocean [17, 18]. However, the biogeochemical and ecosystem consequences of infection could vary among different phytoplankton species. Recent evidence suggests that infection of *E. huxleyi* may actually facilitate aggregation and sinking of particulate carbon into the mesopelagic where it is subsequently respired [12, 15]. This may be a result of increased cellular production of transparent exopolymeric particles (TEP) during infection, which acts to enhance particle aggregation and couple infection with microzooplankton grazing [19].

Due to the ecological significance of *E. huxleyi* blooms and the range of recently developed diagnostic tools for studying infection, *E. huxleyi* and EhVs have emerged as one of the best described systems for understanding the molecular mechanisms of infection in marine eukaryotic microalgae [20]. During infection, EhVs co-opt and rewire cellular lipid biosynthetic machinery, producing viral-specific classes of glycosphingolipids (vGSLs) [11, 16, 21] and betaine-like lipids [22]. Accumulation of these polar lipids during infection occur concomitant with a late stage increase in production of reactive oxygen species (ROS; [23–25]), particularly hydrogen peroxide (H_2_O_2_; [25]), caspase activity, and metacaspase expression [10, 16], ultimately leading to programmed cell death (PCD; [26]) in the form of autophagy [27].

The dynamics of ROS production during viral infection are well described [16, 23, 25]. Little is known, however, about the role(s) of reactive nitrogen species (RNS), such as nitric oxide (NO), in modulating subcellular redox pathways and associated cellular responses. NO is a small, uncharged free radical gas that has been shown to be involved in a myriad of biological functions, including immunity, stress adaptation, and normal growth in all branches of life [28–31]. The foundation for this breadth of action rests on the broad reactivity of NO with various cellular targets, as well as its ability to diffuse across membranes to act both intra- and extra-cellularly. Of particular interest is the role of NO in modifying cellular ROS pools, either via radical–radical reactions or indirectly through the regulation of pro- or anti-oxidant pathways [32–34]. Although the sources(s) of NO synthesis in plants and photosynthetic protists is still unresolved [35], its signaling function during cellular response to diverse biotic and abiotic stressors is well known and summarized in several in-depth reviews [36, 37]. In diatoms, for instance, NO is part of a stress-surveillance system that senses and responds to the toxic polyunsaturated aldehyde decadeinal [38–40].

To date, investigations on the role(s) of NO in *E. huxleyi* physiology have been limited. One study demonstrated *E. huxleyi* growth rate and maximum cell densities increased in response to low levels of exogenously added NO [41]. In addition, we have previously shown that *E. huxleyi* cell lysates possess the enzymatic capacity to produce NO from nitrite and NADH via nitrate reductase [42]. There is, however, a notable gap in our knowledge of the mechanistic roles of this ubiquitous signaling molecule in *E. huxleyi* ecophysiology, including during viral infection. Given EhV infection induces PCD through elevated ROS production, it stands to reason that NO may play an important interactive role in infection dynamics. Here, we demonstrate that elevated intracellular NO production is a hallmark of early-stage lytic viral infection of *E. huxleyi* both in culture and in natural populations in the North Atlantic. Scavenging of intracellular NO leads to a dose-dependent reduction in viral burst sizes, indicating that NO is critical for maximal viral production in lab conditions. Using a novel liposome-enabled electron paramagnetic resonance (EPR) spectroscopy method [42], we also show that elevated extracellular NO is observed during infection. Our work further suggests that intracellular NO production upregulates cellular antioxidant activity during the early stages of infection, keeping the cellular redox state favorable for viral replication.

## Materials and methods

### Culture conditions and viral infections

*Emiliania huxleyi* strain CCMP1516 was obtained from the Provasoli-Guillard National Center for Marine Algae and Microbiota and grown in batch culture in f/2 (minus Si) media at 18 °C on a 14:10 light:dark cycle at a light intensity of 250 µmol m^−2^s ^−1^. Virus strain EhV201 (obtained courtesy of W. Wilson, Marine Biological Association, Plymouth, UK) was propagated in batch cultures of *E. huxleyi* CCMP1516. Viral lysates were passed through a 0.45 µm pore-size PVDF syringe filter to remove cell debris. For infection experiments, *E. huxleyi* was inoculated with EhV during mid-exponential growth (~5.0 × 10^5^ cells ml^−1^) at a virus-to-host ratio of 5:1. Uninfected *E. huxleyi* cultures served as controls.

### Enumeration of cells and viruses

*E. huxleyi* cell abundances were quantified using either a BD InFlux Mariner 209S flow cytometer or a BD Accuri C6 flow cytometer. Cell abundances were determined based on the chlorophyll autofluorescence (E_x_/E_m_: 488 nm, 692 nm) vs. forward scatter signature. Free viruses were quantified by flow cytometry according to [43]. See Supplementary Information for additional flow cytometry methods.

### Intracellular NO detection

Semi-quantitative measurements of intracellular NO were made using the NO specific fluorescent probe DAF-FM Diacetate (DAF-FM DA; Thermo Fisher, Waltham, MA). DAF-FM DA passes through cell membrane, is cleaved by intracellular esterases to DAF-FM, and accumulates inside the cell. DAF-FM is non-fluorescent until it binds to NO or its oxidized products to form a fluorescent triazole product, DAF-FM-T [44]. Stocks of DAF-FM DA were made to 5 mM in DMSO (Sigma-Aldrich, St. Louis, MO) and used at a final concentration of 5 µM. Stained samples were incubated in the dark at RT for 45 min. Mean fluorescence intensity per cell was determined by flow cytometry (E_x_/E_m_: 488 nm, 520 nm). An unstained sample was run to account for background autofluorescence. Several controls were run to contextualize DAF-FM DA results and are described below.

### Chemical identification of DAF-FM-T in cells

The presence of the fluorescent DAF-FM-T triazole product in cells treated with NO donors was chemically confirmed using high performance liquid chromatography (HPLC) and ion-trap mass spectrometry (MS). Cultures of *E. huxleyi* CCMP1516 were treated with the NO donors S-nitroso-N-acetylpenicillamine (SNAP; Thermo Fisher) and sodium nitroprusside (SNP; Sigma-Aldrich) at 100 µM and 1 mM, respectively, and stained with 5 µM DAF-FM DA. A DAF-FM-T standard was generated in vitro by exposing 50 µM DAF-FM (Thermo Fisher) to an excess ( > 50 mM) of SNP. Identification of DAF-FM and DAF-FM-T was confirmed by MS^2^ spectra of the 413 *m/z* and 424 *m/z* molecular ions, which showed diagnostic neutral loss of CO_2_ (44 *m/z*) as previously characterized [45]. See Supplementary Information for full description.

### Intracellular esterase activity

Intracellular esterase activity was measured in infected and uninfected cells using a general fluorogenic esterase substrate. Cell lysates were generated and protein was quantified (see Supplementary Information). A total of 2 µg of protein was incubated with 25 µM 4-Methylumbelliferyl butyrate (Sigma-Aldrich). The time course of fluorescence (E_x_/E_m_: 365 nm, 440 nm) was measured every 2 min for 1 h using a SpectraMax M3 microplate reader. Esterase activity is expressed as the rate of change in fluorescence (RFU) per µg protein.

### Intracellular ROS and cell death analysis

Cellular ROS production was assessed using the fluorescent probe CM-H_2_DCFDA (Thermo Fisher), which has a broad reactivity with a variety of ROS. Stocks of CM-H_2_DCFDA were made up to 1 mM in DMSO and used at a final concentration of 5 µM. Samples were incubated in the dark at RT for 60 min. The percentage of dead cells in cultures was determined using SYTOX Green (Thermo Fisher). SYTOX Green (5 mM stock solution in DMSO) was used at a final concentration of 1 µM and incubated in the dark at RT for 10–15 min. Stained samples (E_x_/E_m_: 488 nm, 520 nm), along with an unstained control, were analyzed by flow cytometry.

### Extracellular NO measurements

In situ, cell-derived NO produced during infection and present in the surrounding media was monitored using liposome-encapsulated spin trap (LEST) and electron paramagnetic resonance (EPR) spectroscopy, as previously described [42]. In brief, liposomes were prepared from a 9:1 molar ratio of the phospholipids POPC and DPPG (Avanti Polar Lipids, Alabaster, AL) in chloroform containing 10 mM of the NO spin trap N-methyl-D-glucamine dithiocarbamate (MGD) and 2 mM ammonium iron (II) sulfate. See Supplementary Information for more details.

LEST (25 µL) was incubated in 10 ml of triplicate infected and uninfected cultures adjusted to equal cell densities with f/2 (minus Si) media for 3 h in the dark at RT. LEST incubated in f/2 (minus Si) served as a negative control; LEST incubated in the presence of 200 µM of the NO donor NOC-9 (Sigma-Aldrich) served as a positive control. After incubation, LEST was pelleted by centrifugation (20,000 × *g*, 30 min, 4 °C). The supernatant was removed such that 30 µL of LEST pellet and buffer remained. The pellet and buffer were homogenized, flash frozen in liquid nitrogen, and stored at −80 °C. For EPR analysis, frozen LEST was thawed and drawn up into microcapillary tubes. EPR spectra were collected and the signal from spin-trapped NO quantified as described previously [42]. See Supplementary Information for more details.

### NO donor, NO scavenger, and hydrogen peroxide treatments

To further investigate the cellular role of NO during infection, the following experiments were conducted: (1) *E. huxleyi*-EhV infection in the presence of an NO scavenger, (2) monitoring physiology of *E. huxleyi* pre-treated with various concentrations of an NO donor and subsequently challenged with hydrogen peroxide (H_2_O_2_), and (3) determination of the total antioxidant capacity of *E. huxleyi* cell lysates both treated with an NO donor and undergoing infection. The NO donor used was S-nitroso-N-acetylpenicillamine (SNAP) and treatments were done at concentrations empirically determined to be non-lethal (up to 100 µM; Figure S7) for at least 16 h prior to H_2_O_2_ treatment or biomass harvest. Given SNAP has a donor half-life of ~6 h, this time period represents >2 half-lives. The NO scavenger used was carboxy-PTIO potassium salt (c-PTIO; Thermo Fisher) and was applied to cells at the time of infection at a range of concentrations (250 µM–1 mM dissolved in MilliQ). Treatments with H_2_O_2_ (30% w/w; Sigma-Aldrich) were performed between 10 and 100 µM. Cell abundance, percent dead cells, intracellular NO and ROS, and the photochemical quantum yield of photosystem II (F_v_/F_m_; see Supplementary Information) were monitored for these experiments.

### Total antioxidant capacity

*E. huxleyi* cell lysates were generated and protein concentration was determined (see Supplementary Information). The total enzymatic and non-enzymatic antioxidant capacity (TAC) of the extracts was determined using the Antioxidant Assay Kit (Cayman Chemical, Ann Arbor, MI), which measures the capacity of cell extracts to prevent the oxidation of ABTS (2,2’-azino-di-[3-ethylbenzthiazoline sulphonate]) in the presence of H_2_O_2_ compared to a standard of the vitamin E analog Trolox (6-hydroxy-2,5,7,8-tetramethylchroman-2-carboxylic acid). The assay and standard curve were run according to the manufacturer’s instructions. Absorbance at 750 nm was measured using a SpectraMax M3 microplate reader. TAC is expressed as the concentration (mM) of antioxidants in equivalents of Trolox normalized to the total protein concentration of the sample.

### Fieldwork

Intracellular NO, ROS, and cell death were assessed for open ocean, EhV-infected *E. huxleyi* populations in the Northeast Atlantic during the North Atlantic Virus Infection of Coccolithophores Expedition (http://www.bco-dmo.org/project/2136) aboard the R/V Knorr. The NA-VICE traversed a 2000 nautical mile transect from the Azores to Iceland and identified *E. huxleyi* blooms at different stages of bloom formation and viral infection [12, 13, 15]. Individual CTD casts were characterized and grouped into stations- “early infection (EI)”, “early infection revisited (EI_R_)”, “late infection (LI)”, and “post infection (PI)”—using a combination of MODIS/AQUA satellite imagery, a suite of diagnostic lipid- and gene-based molecular biomarkers, analytical flow cytometry, in situ optical sensors, and sediment traps (methods described by [12]). Here we further divided the “early infection” population into “EI_1_” and “EI_2_” given the greater number of samples available at this site for analysis and to provide higher temporal resolution to the trends. In order to explore the robustness of the patterns observed, we explored additional data from three CTD casts not analyzed in the aforementioned study [12], along with an individual CTD cast (92) from EI_1_ to illustrate a comparative signal for an early infected population. See Supplementary Information for a list of casts in each station.

Water was collected at six depths—extending from the subsurface, through the mixed layer encompassing the chlorophyll maximum, and down to 150 m—using Niskin bottles mounted on a 24-position rosette equipped with a Seabird SBE conductivity-temperature-depth (CTD) profiler. Sub-samples were stained with DAF-FM Diacetate, CM-H_2_DCFDA, and SYTOX Green (5 µM) as described above. Stained samples, along with an unstained control, were run on a Guava flow cytometer (EMD Millipore, Burlington, MA) in duplicate. We present data from three depths per cast corresponding to the depth at which *E. huxleyi* cell abundance was highest, along with one sampling depth above and one sampling depth below the *E. huxleyi* maximum, in box-and-whisker plots. These depths generally ranged from 8 to 40 m and are listed in Table S2.

### Data analysis and statistics

Flow cytometry data collected for laboratory experiments were analyzed using FlowJo (v. 10.2). Statistics (counts and mean fluorescence) were based on at least 1000 *E. huxleyi* events. Mean fluorescence per cell for DAF-FM Diacetate and CM-H_2_DCFDA stained samples are reported as the difference between the mean 520 nm fluorescence per cell of the stained sample and an unstained control. Percent SYTOX Green positive cells are reported as the percent of the total *E. huxleyi* population that has elevated 520 nm fluorescence relative to an unstained control.

Flow cytometry data for fieldwork were analyzed using GuavaSoft InCyte (v. 2.2.2). *E. huxleyi* was distinguished by pre-gating all events by chlorophyll and gating the *E. huxleyi* population off side scatter and forward scatter signatures corresponding to a reference culture. Statistics (counts and mean fluorescence) were based on at least 50 *E. huxleyi* events, with most samples encompassing 100–400 events, and averaged between two replicates per depth.

Statistically significant differences between infected and uninfected cultures for the parameters measured in this study were determined with Student’s *t*-tests (*p* < 0.05). To test differences between multiple means, a one-way ANOVA with a Tukey HSD post hoc test was used. Error bars on all graphs are ± standard error of the mean (se). Linear regression analysis was used to explore relationships between various parameters in the NA-VICE dataset. All statistical tests were performed in R and plots were generated using the ggplot2 package.

## Results

### Intracellular NO production increases during viral infection

**Fig. 1:**
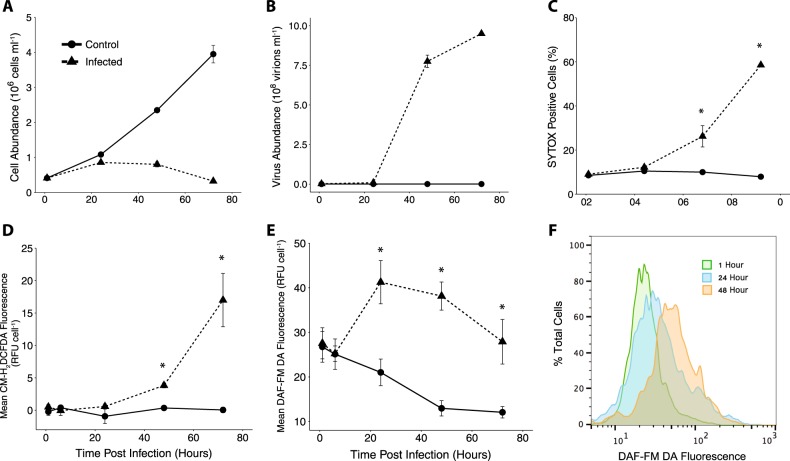
Dynamics of viral infection of *E. huxleyi* CCMP1516 by EhV201. **a** Cell abundance and **b** viral abundance of infected (triangles/dashed line) and uninfected (circles/solid line) cultures are a mean of *n* = 2 (±se) from one representative viral infection experiment. **c** Percent dead cells assessed by SYTOX Green, **d** intracellular ROS assessed by CM-H_2_DCFDA, and **e** intracellular NO assessed by DAF-FM DA. Values represent the mean of at least *n* = 4 (±se) across at least 3 distinct infection experiments. Statistically significant differences between infected and control cultures were determined using unpaired Student’s *t*-test (*p < 0.05). **f** Histogram overlay of DAF-FM DA fluorescence values of one representative infected culture at 1, 24, and 48 hpi

The onset of cell lysis by EhV infection at 48–72 h post infection (hpi) was marked by 12.5 and 60% decreases in cell abundance between 24–48 hpi and 48–72 hpi, respectively (Fig. 1a), coinciding with EhV production (Fig. 1b). Cell decline was concomitant with increases in both the proportion of dead or dying cells indicated by SYTOX Green (26% of culture at 48 hpi and 58% culture at 72 hpi; Fig. 1c) and intracellular ROS indicated by CM-H_2_DCFDA, increasing ~3-fold at 48 hpi and ~17-fold at 72 hpi (Fig. 1d). Both SYTOX and CM-H_2_DCFDA signals were strongest at 72 hpi. Notably, intracellular NO production did not follow these trends. Enhanced intracellular NO, assessed with DAF-FM DA, was observed earlier than the burst of ROS, increasing 1.5- to 2-fold above uninfected controls at 24 hpi (Fig. 1e, f). Intracellular NO production in infected cultures remained elevated compared to uninfected controls throughout the course of infection. A steady decrease in basal intracellular NO production was observed in uninfected control cells over the same time frame (Fig. 1e).

Intracellular esterase activity in infected cells remained unchanged during infection, while control cells displayed an increase in esterase activity during growth (Figure S1), suggesting that changes in intracellular esterase activity were not responsible for the higher DAF-FM DA fluorescence observed in infected cells. Furthermore, treatment of *E. huxleyi* with the NO donor SNAP increased DAF-FM DA cellular fluorescence, while the NO scavenger diminished fluorescence (Figure S2). The presence of the NO-bound, fluorescent DAF-FM-T product was also chemically confirmed using HPLC MS/MS in cells treated with two NO donors in a dose-dependent manner (Figures S3 and S4).

### Viral infection triggers enhanced extracellular NO

**Fig. 2:**
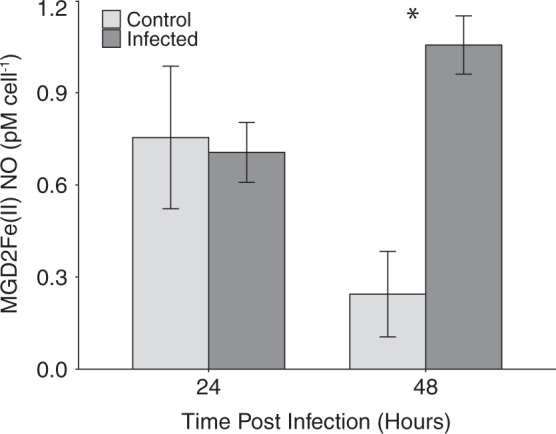
Concentration of extracellular NO in *E. huxleyi* CCMP1516 infected with EhV201 (dark gray bars) and control cultures (light gray bars). Values represent the mean concentration of spin trap bound NO per cell over a 3 h incubation period (*n* = 3, ±se). Statistically significant differences between infected and control cultures were determined using unpaired Student’s *t*-test (*p < 0.05)

Statistically significant differences in extracellular NO production were observed between infected and uninfected cultures at 48 hpi, 24 h after peak intracellular NO production (Fig. 2). Four-fold higher cell-normalized extracellular NO was observed at 48 hpi in the infected cultures relative to uninfected controls. A control experiment in which cells were incubated with LEST for 3 h found no impacts of LEST on cell survival, intracellular NO production, intracellular ROS production, or photochemical quantum yield of photosystem II (Fv/Fm) (Table S1).

### NO scavenging decreases viral burst size

**Fig. 3:**
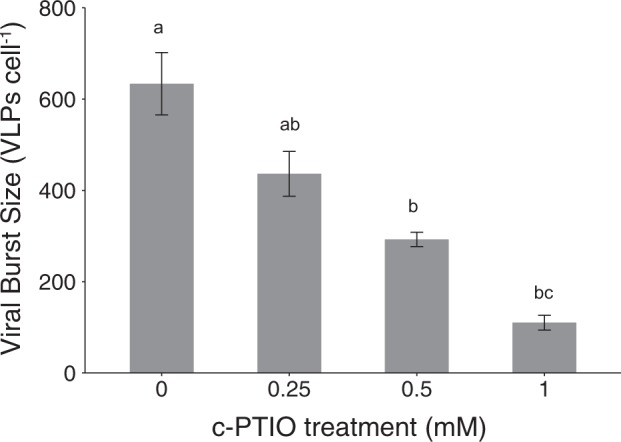
Viral burst sizes of infected *E. huxeyi* CCMP1516 treated with the NO scavenger c-PTIO, represented as the number of viral particles produced per cell lysed between 24 and 72 h post infection. Data are the mean of *n* = 2 (±se) and are a representative subset of multiple experiments. Statistically significant differences were determined using one-way ANOVA with the Tukey HSD post hoc test (letters denote statistically different subgroups; *p* < 0.05)

EhV infection proceeded similarly in cells treated with the NO scavenger c-PTIO as that observed for untreated cells across a range of concentrations, with the onset of cell lysis and viral production occurring 48–72 hpi (Figure S5). There was, however, a statistically significant, dose-dependent decrease in viral burst size (the number of viruses produced per cell lysed between the 24–72 hpi) in c-PTIO-treated cells undergoing infection (Fig. 3). Exposure of uninfected control *E. huxleyi* cells to c-PTIO yielded no significant differences in cellular growth rates or Fv/Fm compared to untreated cells over the course of 72 h (Figure S6).

### NO production stimulates antioxidant activity

**Fig. 4:**
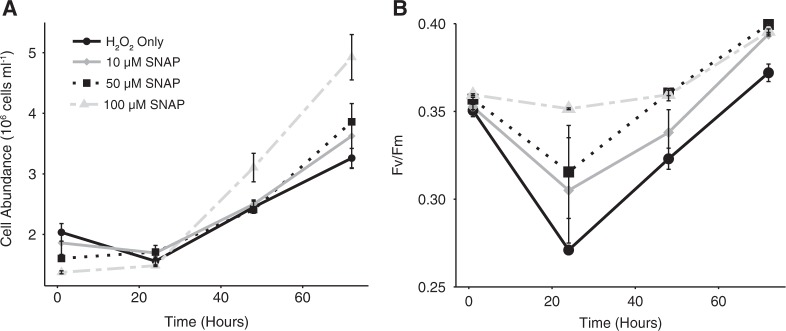
Response of cells pre-treated with exogenous NO to H_2_O_2_. **a** Cell abundance and **b** Fv/Fm of H_2_O_2_ treated (100 µM) *E. huxleyi* CCMP1516 pre-treated with different concentrations (10 µM, 50 µM, and 100 µM) of the NO donor SNAP. Data are a representative subset of multiple experiments and values represent the mean of *n* = 2 (±se)

Cells that were pre-treated with the NO donor SNAP prior to challenge with H_2_O_2_ (100 µM) had enhanced growth compared to cells treated with H_2_O_2_ only over the course of 72 h (Fig. 4a). Additionally, none of the SNAP pre-treated cultures experienced net cell death in the first 24 h of the experiment, though growth rates were diminished (Fig. 4a). In contrast, cells treated with H_2_O_2_ only experienced net cell decline within the first 24 h of treatment. SNAP pre-treated cultures were also able to maintain higher Fv/Fm values over the course of 72 h compared to the H_2_O_2_-only control (Fig. 4b). No effect was observed in DMSO-only pre-treated cultures (SNAP is dissolved in DMSO) in their response to H_2_O_2_ treatment (Figure S8).

**Fig. 5:**
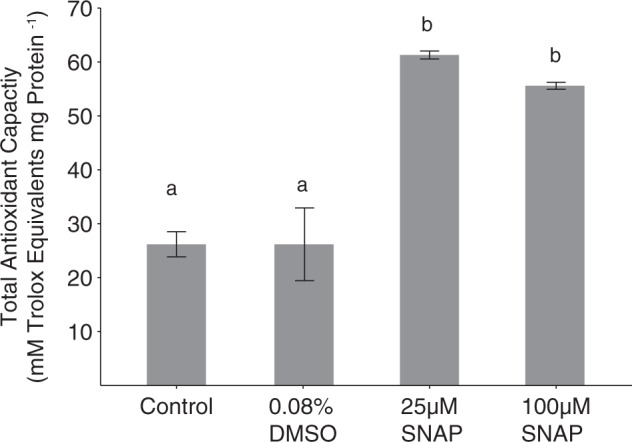
Protein-normalized, cellular antioxidant capacity of lysates from *E. huxleyi* CCMP1516 cultures treated with SNAP, along with a DMSO only and untreated controls. Statistically significant differences were determined using one-way ANOVA with the Tukey HSD post hoc test (letters denote statistically different subgroups, *p* < 0.05). Data shown are a representative subset of multiple experiments and are the mean of *n* = 2 (±se) per treatment

**Fig. 6:**
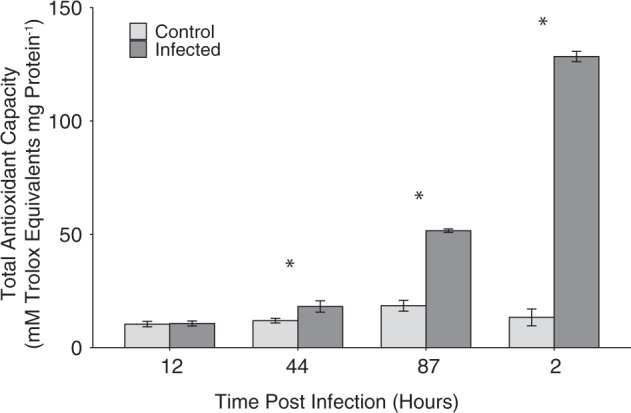
Protein-normalized, cellular antioxidant capacity of lysates from *E. huxleyi* CCMP1516 cultures undergoing infection with EhV201 (dark gray bars) and uninfected control cultures (light gray bars). Values represent the mean of *n* = 5 (+/− se) pooled from biological duplicates from one experiment. Statistically significance between control and infected cultures was determined used a Student’s *t*-test (*p < 0.05)

Cell lysates from cultures treated with SNAP exhibited significantly elevated protein-normalized total enzymatic and non-enzymatic antioxidant capacity (TAC) (Fig. 5). The protein-normalized TAC of cells also increased in cells undergoing viral infection, with statistically significant differences between infected and uninfected cells beginning at 24 hpi. Little to no change was detected in uninfected control cultures during this same time period (Fig. 6).

### NO, ROS, and cell death in natural *E. huxleyi* blooms

**Fig. 7:**
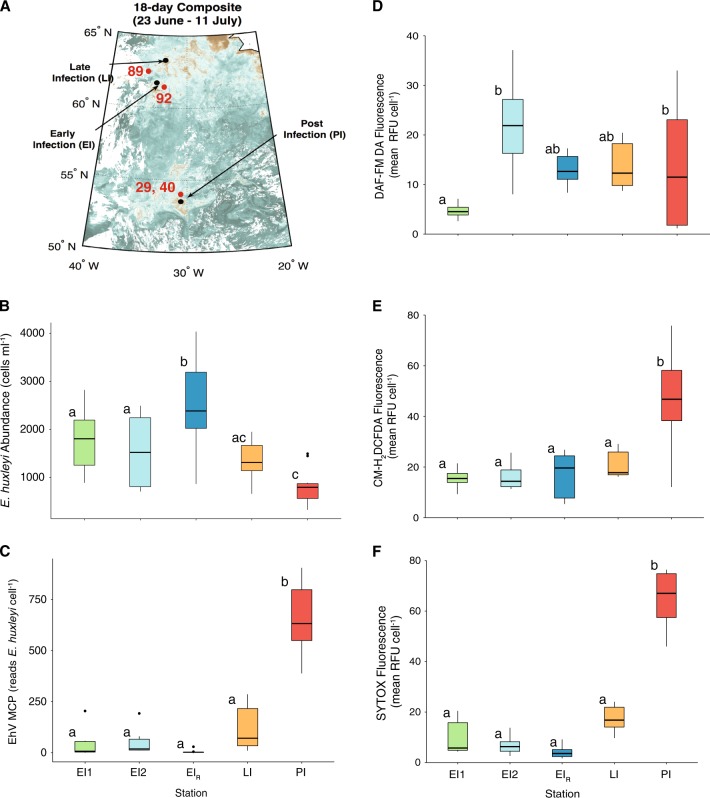
Assessment of diagnostic stains for natural *E. huxleyi* populations in the North Atlantic. **a** Locations of distinct water masses sampled on the NA-VICE cruise along a 2000 nautical mile transect in the North Atlantic. Early, late, and post infection populations were previously characterized using lipid- and gene-based biomarkers [12]. Red numbers 29, 40, 89, and 92 represent CTD casts for which additional analyses are performed in this study. **b**, **c** Box-and-whisker plots showing the respective abundances of host *E. huxleyi* cells (cells ml^−1^) and replicating EhVs (MCP gene copies *E. huxleyi* cell^−1^) for the different sampled populations at three depths where *E. huxleyi* were present in highest abundance. **d**–**f** Box-and-whisker plots showing corresponding DAF-FM DA, CM-H_2_DCFDA, and SYTOX Green fluorescence for these populations. Data are an average of two replicates per depth sampled. Data in (**a**, **b**, **c**, and **f**) were published by Laber et al. [12]. For all box plots, upper and lower bounds of the box represent the 25% and 75% quartiles around the median. Vertical lines extend to data points no greater than 1.5 times the inter-quartile range. Data points that extend beyond this range are represented by dots. Statistically significant differences were determined using one-way ANOVA with the Tukey HSD post hoc test (letters denote statistically different subgroups, *p* < 0.05)

Open ocean *E. huxleyi* blooms were encountered during the NA-VICE cruise in the eastern North Atlantic that were at different stages of viral infection ([12, 13, 15]; Fig. 7a–c). Stations were designated as “early infection” (EI_1_, EI_2_, EI_R_), “late infection” (LI), and “post infection” (PI) by [12] based on the relative abundance of *E. huxleyi* (Fig. 7b) and cell-associated EhV populations (i.e., the number of copies of major capsid protein (MCP) per host cell) (Fig. 7c), as well as the inventories of diagnostic glycosphingolipid (such as vGSLs and sGSLs) and betaine-like lipid biomarkers, all of which are indicative of these stages of infection ([11, 16, 22, 46]; see Discussion). The abundance of *E. huxleyi* cells ranged from ~1000–3000 cells ml^−1^ in early infection stations and decreased in late (~1300 cells ml^−1^) and post (~800 cells ml^−1^) infection stations (Fig. 7b). Early infection stations had low copy numbers of EhV-derived MCP (mean of 44 copies cell^−1^). MCP copy number increased in late (mean of 120 copies cell^−1^) and post (mean of 800 copies cell^−1^) infection (Fig. 7c), indicative of an increased degree of infection.

Intracellular NO production was elevated in EI_2_, EI_R_, LI, and PI, relative to the initial occupation of EI_1_, with a 5.2, 2.8, 3.0, and 5.0-fold higher mean per cell DAF-FM DA fluorescence, respectively (Fig. 7d). Intracellular ROS of *E. huxleyi* at these stations was generally low and not statistically different from each other. ROS was elevated only in *E. huxleyi* cells found in the PI populations (Fig. 7e), with an average 3.5-fold higher mean per cell CM-H_2_DCFDA fluorescence than the other stations, consistent with observations of a late phase infected culture (Fig. 1). Similarly, cell death was moderately elevated in LI populations and significantly higher in PI populations; *E. huxleyi* cells averaged about 6-fold higher mean per cell SYTOX Green fluorescence in PI casts (Fig. 7f) above cells in early infection populations. Taken together, the levels of cellular NO, ROS, and death of *E. huxleyi* cells undergoing different stages of infection generally reflected the patterns observed in lab cultures (Fig. 1).

**Fig. 8:**
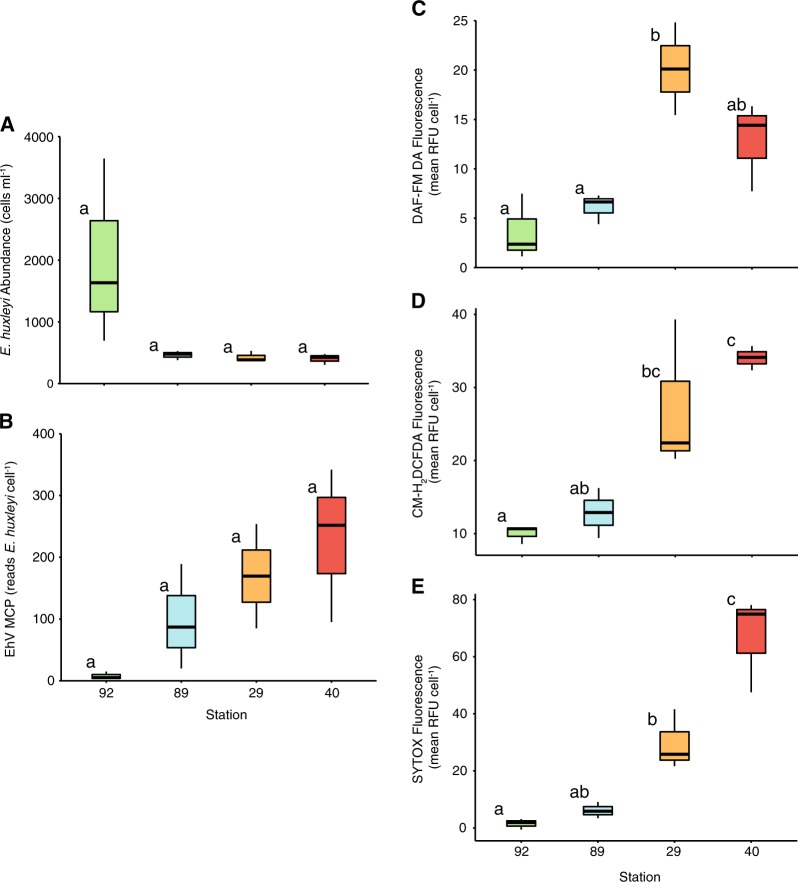
Gene- and fluorescence-based biomarkers for four additional CTD casts conducted during the NA-VICE cruise. **a**, **b** Box-and-whisker plots showing *E. huxleyi* cell abundance (cells ml^−1^) and EhV-derived MCP gene copies *E. huxleyi* cell^−1^ for these casts at three depths where *E. huxleyi* were present in highest abundance. **c**–**e** Box-and-whisker plots showing corresponding DAF-FM DA, CM-H_2_DCFDA, and SYTOX Green fluorescence for these casts. Data are an average of two replicates per depth. For all box plots, upper and lower bounds of the box represent the 25% and 75% quartiles around the median. Vertical lines extend to data points no greater than 1.5 times the inter-quartile range. Statistically significant differences were determined using one-way ANOVA with the Tukey HSD post hoc test (letters denote statistically different subgroups, *p* < 0.05)

These diagnostic parameters were examined for three additional *E. huxleyi* populations (CTD 29, 40, and 89) that were outside of the aforementioned characterized water masses. An individual cast (CTD 92) from the collection of casts performed and characterized at EI_1_ with DNA- and lipid-based biomarkers was also included for comparison (Fig. 8; [12]). While not all biomarkers showed statistically significant differences, likely due to low sample size per station, trends in the data nonetheless suggest that these populations were in distinct phases of infection. CTD casts 29 and 40 appeared most similar to a late infection or post infection scenario, respectively, with high ROS, NO, and cell death signatures. Specifically, CTD cast 29 had median DAF-FM DA, CM-H_2_DCFDA, and SYTOX Green fluorescence values of 20.1, 22.4, and 25.8 RFU, respectively. CTD cast 40 had median DAF-FM DA, CM-H_2_DCFDA, and SYTOX Green fluorescence values of 14.1, 34.1, and 74.9 RFU, respectively. These casts also had high EhV-derived MCP copies *E. huxleyi* cell^−1^ (mean of 170 and 230 copies cell^−1^ for cast 29 and 40), and low host cell abundance (mean less than 450 cells ml^−1^). Cast 29 also had a high vGSL:sGSL ratio (mean ratio of 2.4 across all depths, with a log_10_ depth integrated inventory ratio of −0.042), further indicating active viral infection.

On the other hand, *E. huxleyi* populations sampled at CTD casts 89 and 92 were characterized by lower ROS, NO, and cell death signatures. Cast 89 had median DAF-FM DA, CM-H_2_DCFDA, and SYTOX Green fluorescence values of 6.7, 12.9, and 5.6 RFU, respectively. CTD cast 89 also had moderate EhV-derived MCP copies (mean of 98 copies cell^−1^) and low cell abundance (mean of 460 cells ml^−1^), along with a much lower vGSL:sGSL ratio (mean ratio of 0.60 across all depths, with a log10 depth integrated inventory of −0.35). Cells at CTD cast 92 had the lowest comparative DAF-FM DA, CM-H_2_DCFDA, and SYTOX Green fluorescence with median values of 2.4, 10.7, and 1.9 RFU, respectively. These populations also had very low incidence of EhV-derived MCP (mean of only 8 copies cell^−1^) and high host cell abundance (~2000 cells ml^−1^).

**Fig. 9:**
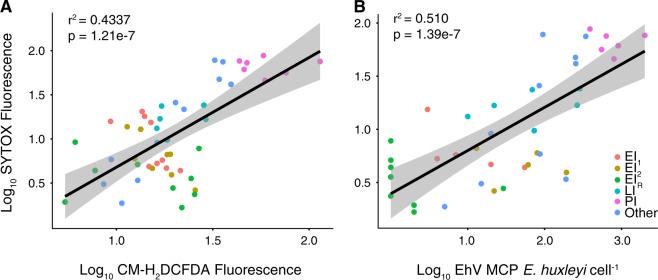
Linear regression analysis of various diagnostic parameters measured across the NA-VICE cruise. **a** Regression of log_10_-transformed mean SYTOX fluorescence vs. log_10_-transformed mean CM-H_2_DCFDA fluorescence. **b** Regression of log_10_-transformed EhV-derived MCP gene copy per *E. huxleyi* cell vs. log_10_-transformed mean SYTOX fluorescence. Shading indicates 95% confidence interval for the regression line

Relationships among these diagnostic stains across the cruise were explored using linear regression analysis. There was a significant positive correlation (*r*^2^ = 0.4337, *p* = 1.21e^−7^) between the levels of intracellular ROS and cell death (mean SYTOX fluorescence) in *E. huxleyi* cells across all CTD casts (Fig. 9a). In addition, there was a positive correlation (*r*^2^ = 0.510, *p* = 1.39e^−7^) between cell death and EhV-derived MCP copies *E. huxleyi* cell^−1^ (Fig. 9b). There were weak or non-significant relationships observed between intracellular NO and: intracellular ROS (*r*^2^ = 0.0696, *p* = 0.0266); cell death (*r*^2^ = −0.0049, *p* = 0.386); and EhV-derived MCP copies *E. huxleyi* cell^−1^ (*r*^2^ = −0.00684, *p* = 0.414) across the stations.

## Discussion

The production of reactive oxygen species (ROS)—radical and non-radical molecules known to have toxic cellular effects such as hydrogen peroxide (H_2_O_2_), superoxide (O_2_^.−^), and the hydroxyl radical (HO^.^)—is a well-documented feature of lytic viral infection in *E. huxleyi* [16, 23, 25]. Unlike in host-pathogen systems of higher plants in which a ROS burst often occurs rapidly following pathogen invasion in order to prevent the spread of infection [47], cellular ROS production in the *E. huxleyi*-EhV system is not observed until late stages of infection occurring at the onset of lysis.These ROS, specifically H_2_O_2_, appear to be required for the induction of the PCD cascade in the host and subsequent cell lysis [25].

Little is known about the function of reactive nitrogen species, such as NO, in *E. huxleyi* physiology or in the emerging picture of the molecular pathways governing viral infection. Our previous work demonstrated that *E. huxleyi* cell lysates possess the ability to produce NO via nitrate reductase (NR) and hinted at the possibility of elevated NO production during infection [42]. It remains unclear, however, whether cellular nitrite levels reach sufficient concentrations during infection to drive NR-dependent NO production. Here, we demonstrate that enhanced intracellular NO production is a hallmark of viral infection in this species and does not occur simultaneously with the accumulation of ROS, indicating an independent function. Statistically significant differences in intracellular NO between infected and non-infected control cells were seen as early as 24 hpi, when there is ~2-fold higher DAF-FM DA fluorescence in infected cells. NO in cells remains elevated throughout the course of infection, relative to uninfected controls. Additionally, we detected an increase in extracellular NO in cultures during infection. These results provide an interesting framework for further exploring the role of NO in viral infection within a population. The ability of NO to act as a diffusible extracellular signal has been previously demonstrated in diatoms where it was shown to be a critical component in the stress perception of *Phaeodactylum tricornutum* [40]. Given our results, it is conceivable that NO produced during viral infection of *E. huxleyi* may serve a similar extracellular signaling role, possibly communicating infection to neighboring cells.

Both the early production and apparent requirement of NO for optimal viral production does suggest a potential cyto-protective role. NO has been shown in both plants and algae to have a broad antioxidant function, allowing cells to cope with various stressors that elicit ROS [48]. For example, NO has been implicated in the response of *Chlorella vulgarus* to copper stress [49], protection of *Scenedesmus obliquus* against H_2_O_2_ [50], and reduction of UV-B damage in the cyanobacterium *Spirulina platensis* [51]. Our findings show that NO may be responsible for similar antioxidant activity in *E. huxleyi*. Pre-treatment of cells with an NO donor increased survival upon subsequent challenge with H_2_O_2_, the main ROS produced during infection. Additionally, exogenously added NO led to an increase in the ability of *E. huxleyi* cell lysates to detoxify H_2_O_2_, a feature of cells also undergoing viral infection.

The antioxidant function of NO in early infected *E. huxleyi* supports previous observations of antioxidant changes that occur in this system. Sheyn et al. [25] showed that significant changes in the expression of antioxidant related genes and metabolites occur in the early stages of infection. Specifically, despite downregulation of some genes involved in ROS detoxification (such as ascorbate peroxidase and catalase) and upregulation of others, net H_2_O_2_ accumulation and cell death induction was not observed until late stages of the infection. *E. huxleyi* is also able to maintain high levels of both total and reduced glutathione pools [25], suggesting a maintenance of antioxidant capacity. Additionally, viral infection has been shown to induce production of the volatile organic sulfur compound dimethyl sulfide (DMS), along with its byproduct acrylic acid [52], both of which are believed to also have an antioxidant function [52, 53]. Therefore, there is evidence to support our observation that increased antioxidant function is a hallmark of infection, and we suggest that that NO may be crucial player in this induction.

Surprisingly, we observed a continued enhancement of cellular antioxidant capacity well into the late stages of infection, when oxidative stress and cell death became apparent. Previous work has demonstrated that prior exposure of the green alga *Chlamydomonas reinhardtii* and the dinoflagellate *Peridinium gatunense* to H_2_O_2_ increases both cellular antioxidant enzyme activity and the cell’s ability to detoxify ROS, but, paradoxically, it also increases sensitivity to subsequent sub-lethal doses of ROS [54]. This sensitivity has been attributed to accumulation of certain metabolites of antioxidant pathways during the initial stress, specifically the metabolite dehydroascorbate [54, 55], which acts as a stress-surveillance system. It is feasible that a similar mechanism occurs in *E. huxleyi* during viral infection, in which increased NO production and antioxidant capacity during the early stages of infection act to sensitize cells to oxidative stress and ROS-induced PCD later on.

The mechanism(s) by which NO production may lead to increased cellular antioxidant capacity in algae is unknown. However, work in plant systems suggest that a major mechanism of NO function is by post-translational modification of antioxidant proteins, particularly s-nitrosylation of cysteine residues [32]. For example, it has been shown that NO binds to the ascorbate peroxidase of *Arabidopsis thaliana*, upregulating its H_2_O_2_-scavenging activity during stress [56]. Other possible points of NO involvement during viral infection include regulation of metacaspases, the activity of which has been demonstrated to be essential for viral infection of *E. huxleyi* [10]. In *Arabidopsis*, for example, NO is a critical regulator of type-II metacaspase 9 [57]. Future work in the *E. huxleyi*-EhV system should explore potential host and/or viral proteins that are targets of NO-mediated post-translational modification in order to get a complete understanding of the role of NO in infection and the antioxidant changes that occur. Our observation that extracellular NO does not accumulate until 48 hpi (24 h after intracellular increases are seen) does point to the existence of intracellular NO sinks during infection.

Our findings also demonstrate NO production by naturally occurring *E. huxleyi* populations undergoing various stages of viral infection in the eastern North Atlantic [12, 13, 15]. These populations were characterized as either early, late, or post infection based on an array of diagnostic lipid (glycosphingolipid and betaine-like lipids) and gene-based (EhV-derived MCP) biomarkers, along with the abundance of host *E. huxleyi* [12, 15]. We were able to ground-truth the diagnostic stains used in our lab cultures in natural populations across a dynamic range of infection states. We observed that intracellular ROS production and cell death generally show similar trends in the field to those in laboratory (Fig. 1; [23–25]) and mesocosm [16] studies. Both are significantly elevated only in the post infection scenario where the abundance of replicating EhVs was high, *E. huxleyi* abundance was low, and lipid biomarkers indicated active infection. When all stations are taken together, a statistically significant linear relationship between cell death and intracellular ROS, as well as between cell death and EhV copy number, is observed, supporting multiple laboratory studies showing that the accumulation of ROS within cells occurs concurrently with initiation of cell death.

Patterns of intracellular NO in *E. huxleyi* at these stations also support our lab-based results. Elevated NO occured in relatively early infection and remains elevated in *E. huxleyi* cells encountered at both the late and post infection scenarios. We identified three distinct early infection phases where EhV-derived MCP copy number was low and *E. huxleyi* cell concentrations were high: EI_1_, EI_2_, and EI_R_. Populations at EI_2_ and EI_R_ were characterized by elevated NO production relative to EI_1_. It may be that the populations sampled at the EI_1_ were early enough in infection that virus-induced NO production had not yet occurred, as illustrated by CTD cast 92.

Finally, data from additional casts reinforced the robustness and predictive power of the relationships that emerged between diagnostic stain data and other established biomarkers of viral infection. The combination of very low levels of cellular NO, ROS, death, virus-specific lipid signatures (vGSL:sGSL), and EhV-derived MCP copy numbers per *E. huxleyi* cell at CTD casts 89 and 92 was indicative infection was either in its beginning stages or was occurring at a low level at these two casts. Furthermore, the relatively high abundances of *E. huxleyi* cells found in CTD cast 92 further suggests that this was a relatively healthy bloom with little to no lytic viral infection occurring. *E. huxleyi* at CTD casts 29 and 40 were marked by relatively high levels of intracellular NO, ROS, and cell death in resident *E. huxleyi* populations. As expected, these populations showed strong evidence of a late or post stage viral infection with high virus-specific lipids (cast 29), high EhV-derived MCP reads, and low abundances of *E. huxleyi*.

Fluorescent dyes targeting cellular lipids have been previously used to quickly and efficiently diagnose viral infection in lab studies [24]. Our field observations suggest that NO production, determined through the DAF-FM DA staining, can also be a useful early indicator of the onset of lytic viral infection in natural *E. huxleyi* populations. NO can be assessed in real-time and its increase occurs prior to the emergence of other fluorescent signatures and biomarkers, many of which can only be analyzed on shore. Combined with classic and previously established infection biomarkers, fluorescent intracellular NO, ROS, and death indicators can provide an in situ, high-resolution assessment of the stage of lytic viral infection and physiological status of natural blooms.

## Conclusion

Our work implicates the free radical NO as a crucial player in the molecular pathways governing the viral infection of *E. huxleyi*, distinct from ROS production. Furthermore, intracellular NO may have an antioxidant function, keeping ROS accumulation low so that viruses can replicate and assemble in a redox favorable environment. The application of an exogenous NO donor to *E. huxleyi* cultures enhanced survival in the face of subsequent H_2_O_2_ stress. Similarly, cultures undergoing infection and treated with a low dose of exogenous NO exhibited enhanced ability to detoxify H_2_O_2_. Additionally, the patterns of NO production, ROS production, and cell death seen in the laboratory were observed across a dynamic range of infection states for natural *E. huxleyi* populations sampled in the North Atlantic. Taken together, our culture studies and fieldwork demonstrate that the use of this suite of stains, along with classic lipid- and gene-based biomarkers, helps to more fully describe and diagnose viral infection status in natural *E*. *huxleyi* populations.

## Electronic supplementary material

Supplementary Information
