# Supplementary material for: Nitric oxide production and antioxidant function during viral infection of the coccolithophore Emiliania huxleyi
Source: ISME J. 2019 Jan 3;13(4):1019–31. doi: 10.1038/s41396-018-0325-4 (PMC6461841; doi:10.1038/s41396-018-0325-4)
Supplement: Supplementary file 1 — Supplementary Information [file 41396_2018_325_MOESM1_ESM.docx]

**Supplementary Information**

**1. Full Materials and Methods**

*Culture conditions and viral infections*

*Emiliania huxleyi* strain CCMP1516 was obtained from the Provasoli-Guillard National Center for Marine Algae and Microbiota and grown in batch culture in f/2 (minus Si) media at 18 ^o^C on a 14:10 light:dark cycle at a light intensity of 250 µmol m^-2^ s^-1^. Virus infections were done using strain EhV201 (obtained courtesy of W. Wilson, Marine Biological Association, Plymouth, UK) propagated in batch cultures of *E. huxleyi* CCMP1516. Viral lysates were passed through a 0.45 µm pore-size PVDF syringe filter to separate from cellular debris. For infection experiments, *E. huxleyi* were inoculated with EhV during mid-exponential growth (~5.0 x 10^5^ cells mL^-1^) at a virus to host ratio of 5:1. Uninfected *E. huxleyi* cultures served as controls.

*Enumeration of cells and viruses*

*E. huxleyi* cell abundances were quantified using either a BD InFlux Mariner 209S flow cytometer or a BD Accuri C6 flow cytometer, both equipped with a 488 nm laser. Cell abundances were determined based on the chlorophyll autofluorescence (E_x_/E_m_: 488 nm, 692 nm) vs. forward scatter (FSC) signature typical for *E. huxleyi*. Typical flow rates used with the BD InFlux ranged from 10 µL min^-1^ to 25 µL min^-1^. The flow rate on the BD Accuri C6 was set to slow (14 µL min^-1^). At least 1000 *E. huxleyi* cells were sampled per replicate for cell abundance determination, as well as for the fluorescence measurements described below.

Free viruses were quantified using a BD InFlux Mariner 209S flow cytometer according to (43). Samples were fixed with 0.5% glutaraldehyde (Sigma-Aldrich, St. Louis, MO), flash frozen in liquid nitrogen, and stored at -80^o^C. Samples were then thawed, diluted 1:50 with Tris-EDTA buffer containing SYBR Gold (Thermo Fisher, Waltham, MA) at a dilution of 1:20,000 of the commercial stock, and heated for 10 min at 80 ^o^C. The EhV population was gated and enumerated based on the 520 nm fluorescence vs. side scatter (SSC) signature. Typical flow rates used for enumeration ranged from 10 µL min^-1^ to 25 µL min^-1^ and samples were collected from 15-30 s.

*Intracellular NO detection*

Semi-quantitative measurements of intracellular NO in *E. huxleyi* were made using the NO specific fluorescent probe DAF-FM Diacetate (Thermo Fisher). DAF-FM Diacetate passes through cell membranes, is cleaved by intracellular esterases to DAF-FM which then accumulates inside the cell. DAF-FM is non-fluorescent until it binds to NO or its oxidized products to form the fluorescent triazole product, DAF-FM-T (44). Stocks of DAF-FM Diacetate were made to 5 mM in DMSO (Sigma-Aldrich) and used at a final concentration of 5 µM. Stained samples were incubated in the dark at RT for 45 min. The mean fluorescence intensity per cell was then determined by flow cytometry (E_x_/E_m_: 488 nm, 520 nm). For each stained sample a corresponding unstained sample was run to account for background autofluorescence. Several controls were run to contextualize DAF-FM DA results and are described below.

*Chemical identification of DAF-FM-T in cells*

The presence of the fluorescent DAF-FM-T triazole product in cells treated with NO donors was chemically confirmed by using high performance liquid chromatography (HPLC) and ion-trap mass spectrometry (MS). Cultures of *E. huxleyi* CCMP1516 were treated with both S-nitroso-N-acetylpenicillamine (SNAP; Thermo Fisher) and sodium nitroprusside (SNP; Sigma-Aldrich) at 100 µM and 1 mM, respectively, and stained with 5 µM DAF-FM DA. After 1 h incubation, cells were pelleted by centrifugation (20,000g, 15 min, 4 ^o^C), resuspended in MilliQ, and sonicated (3 X 30 sec on ice, power setting 1, Microson ultrasonic cell disrupter; Misonix, Farmingdale, NY) followed by flash freezing in liquid nitrogen. Lysed samples were then centrifuged again and the supernatant analyzed.

HPLC was performed using a Novapak C18 (4 μm, 3.9 x 150 mm; Waters) column with two eluents at a constant flow rate of 500 µL min^-1^: A = water, B = 70:30 acetonitrile:isopropanol. Both eluents also contained 0.1% acetic acid and 1% 1M ammonium acetate. The eluent gradient was from 95% A to 30% A over 15 minutes with an equilibration period of 5 min at 95% A at the end of the run. A Thermo LCQ Fleet ion-trap mass spectrometer was used with a heated- electrospray source with the following settings: source temp 350ºC, capillary temp 250ºC, sheath and auxiliary gas flows 20 and 15 respectively (arbitrary units), source voltage 4.5kV. Source conditions were determined by tuning while infusing a solution of the DAF-FM and DAF-FM-T analytes. Positive ionization was used, though both analytes yielded suitable signals in both positive and negative ion modes. Under these conditions retention times of DAF-FM and DAF-FM-T were 16.4 and 11.9 minutes respectively. Molecular ions were observed as expected at 413 m/z for DAF-FM and 424m/z for DAF-FM-T. The mass spectrometer was operated in full scan mode from 100-1500m/z, though extracted ion chromatograms were used for quantitation. A DAF-FM-T standard was generated by exposing 50 µM DAF-FM (Thermo Fisher) to an excess (>50mM) of the NO donor sodium nitroprusside (SNP). Quantitation of DAF-FM-T in cell extracts was achieved by external calibration with DAF-FM-T standard solutions between 0.2 to 8 µM (10 µL injections) and a linear response was observed for DAF-FM-T between 2 and 80 pmol on column. The limit of detection, as defined by a 3:1 signal to noise ratio, was determined to be 2 pmol on column. Identification of DAF-FM and DAF-FM-T was confirmed by MS^2^ spectra of the 413 m/z and 424 m/z molecular ions, which showed diagnostic neutral loss of CO_2_ (44 m/z) as previously characterized (45).

*Intracellular esterase activity*

Intracellular esterase activity was measured in infected and uninfected cells using a general esterase fluorogenic substrate. To generate cell lysates, *E. huxleyi* biomass was harvested by filtering 50-125 ml of cell culture onto 1.2 µm pore-size Isopore filters (RTTP; EMD Millipore) under low vacuum (<20 kPa). Filters were immediately flash frozen in liquid nitrogen and stored at -80^o^C until analysis. Biomass was resuspended in PBS (5 mM potassium phosphate, pH 7.4, containing 0.9% sodium chloride) and lysed by sonication (3 X 30 sec on ice, power setting 1; Microson ultrasonic cell disrupter) followed by flash freezing in liquid nitrogen. Lysates were centrifuged (20,000g, 15 min, 4^o^C) and the supernatant was retained. Protein in lysates was quantified with the DC protein assay kit, following the manufacturer’s instructions (Bio-Rad, Hercules, CA), using standards of bovine serum albumin (Thermo Fisher) diluted in PBS. Absorbance at 750 nm was read using a SpectraMax M3 microplate reader (Molecular Devices).

# Intracellular esterase activity was measured by incubating cell lysates containing a total of 2 µg of protein with 25 µM 4-Methylumbelliferyl butyrate (Sigma-Aldrich). Fluorescence (E_x_/E_m_: 365 nm, 440 nm) was measured using a SpectraMax M3 microplate reader every 2 min for 1 h. Esterase activity was expressed as the rate of change in fluorescence (RFU) per µg protein. Fresh standards of MUF (Sigma-Aldrich) were run daily to ensure the linear relationship between free MUF and 440 nm fluorescence between a concentration of 25 µM and 0.025 µM.

*Intracellular ROS and cell death analysis*

Cellular ROS production was assessed using the fluorescent probe CM-H_2_DCFDA (Thermo Fisher), which has a broad reactivity with a variety of radical and non-radical ROS. Stocks of CM-H_2_DCFDA were made up to 1 mM in DMSO and used at a final concentration of 5 µM. Samples were incubated in the dark at RT for 60 min. The percentage of dead cells in cultures was determined using SYTOX Green (Thermo Fisher). SYTOX Green (5 mM stock solution in DMSO) was used at a final concentration of 1 µM. Samples were incubated in the dark at RT for 10-15 min. Stained samples (E_x_/E_m_: 488 nm, 520 nm), along with an unstained control, were analyzed by flow cytometry.

*Extracellular NO measurements*

*In situ*, cell-derived NO produced during infection and present in the surrounding media was monitored using liposome-encapsulated spin trap (LEST) and electron paramagnetic resonance (EPR) spectroscopy, as previously described (42). In brief, liposomes were prepared from a 9:1 molar ratio of the phospholipids 1-palmitoyl-2-oleoyl-sn-glycero-3-phosphocholine and 1,2-dipalmitoyl-*sn*-glycero-3-phospho-(1′-*rac*-glycerol), POPC and DPPG respectively, in chloroform (Avanti Polar Lipids, Alabaster, AL). A lipid film was formed by rotary evaporation and dried overnight under vacuum. The lipid film was suspended in buffer containing 10 mM of the spin trap N-methyl-D-glucamine dithiocarbamate (MGD) and 2mM ammonium iron(II) sulfate in a ratio of 1 mL buffer to 100 mg lipid mixture. The resulting multilamellar vescivles (MLVs) were freeze-thawed (x 5 cycles) in liquid nitrogen and stored in liquid nitrogen. Prior to use, the MLVs were suspended in HEPES buffer (20 mM, 140 mM NaCl, pH 7.4) and filtered through a PD-10 desalting column (GE Life Sciences, Chicago, IL) to remove extra-liposomal MGD and iron, yielding LEST.

LEST (25 µL) was incubated in 10 ml of triplicate infected and uninfected cultures adjusted to equal cell densities with f/2 (minus Si) media for 3 h in the dark at RT. LEST incubated in f/2 (minus Si) served as a negative control; LEST incubated in the presence of 200 µM of the NO donor NOC-9 (Sigma-Aldrich) served as a positive control. After incubation, LEST was pelleted by centrifugation (20,000g, 30 min, 4 ^o^C). The supernatant was removed such that 30 µL of LEST pellet and buffer remained. The pellet and buffer were homogenized, flash frozen in liquid nitrogen, and stored at -80 ^o^C until analysis. For EPR analysis, frozen LEST was thawed and drawn up into microcapillary tubes. EPR spectra were collected and the signal from spin-trapped NO quantified as described previously (41). In brief, continuous-wave EPR spectra were collected at X-band, 9.8 GHz, with a Bruker EMXPlus EPR spectrometer with the standard high sensitivity X-band resonator. MGD_2_Fe(II)-NO was quantified by comparing spectra peak areas or peak-to-trough heights to a standard generated from the stable nitroxide radical TEMPOL (Sigma-Aldrich) of a known concentration.

To test whether incubation with LEST had cytotoxic effects on cells, a suite of physiological measurements were taken before and after cells were incubated at RT in the dark for 3 h and compared to a control culture which had no LEST addition. These measurements included intracellular NO, intracellular ROS, % dead cells, cell abundance as well as the photochemical quantum yield of photosystem II (F_v_/F_m_) and functional cross-section of photosystem II (sigma). Photosynthetic parameters were measured using a custom-built fast Fluorescence Induction and Relaxation System (59).

*NO donor, NO scavenger, and hydrogen peroxide treatments*

To further investigate the cellular role of NO, the following experiments were conducted: (1) *E. huxleyi* infection in the presence of an NO scavenger, (2) monitoring physiology of *E. huxleyi* pre-treated with various concentrations of an NO donor and subsequently challenged with hydrogen peroxide (H_2_O_2_), and (3) determination of the total antioxidant capacity of *E. huxleyi* treated with an NO donor and undergoing infection. The NO donor used was S-nitroso-N-acetylpenicillamine (SNAP) and treatments were done at concentrations empirically determined to be non-lethal (less than 250 µM; data not shown) for at least 16 h. Given SNAP has a donor half-life of ~6 h, this time period represents >2 half-lives. The NO scavenger used was carboxy-PTIO potassium salt (c-PTIO; Thermo Fisher) and was applied to cells at the time of infection (T_0_) at a range of concentrations (250 µM – 1 mM dissolved in MilliQ). Treatments with H_2_O_2_ (30% w/w; Sigma-Aldrich) were performed between 10 – 100 µM**.** Cell abundance, percent dead cells, intracellular NO and ROS, and the photochemical quantum yield of photosystem II (F_v_/F_m_) were monitored for these experiments. Photosynthetic parameters were measured using a custom-built fast Fluorescence Induction and Relaxation System (59).

*Total antioxidant capacity*

*E. huxleyi* lysates were generated and protein concentration was determined as described preivously. The total enzymatic and non-enzymatic antioxidant capacity (TAC) of the extracts was determined using the Antioxidant Assay Kit (Cayman Chemical, Ann Arbor, MI), which measures the capacity of cell extracts to prevent the oxidation of ABTS (2,2'-azino-di-[3-ethylbenzthiazoline sulphonate) in the presence of H_2_O_2_ compared to a standard of the vitamin E analog, Trolox (6-hydroxy-2,5,7,8-tetramethylchroman-2-carboxylic acid). The assay and standard curve were run according to the manufacturer’s instructions. Absorbance at 750 nm was measured using a SpectraMax M3 microplate reader. TAC is expressed as the concentration (mM) of antioxidants in equivalents of Trolox normalized to the total protein concentration of the sample.

*Fieldwork*

Intracellular NO, ROS, and cell death were assessed for open ocean, EhV-infected *E. huxleyi* populations in the Northeast Atlantic during the *North Atlantic Virus Infection of Coccolithophore Expedition* (<http://www.bco-dmo.org/project/2136>) aboard the *R/V Knorr*. The NA-VICE traversed a 2000 nautical mile transect from the Azores to Iceland and identified *E. huxleyi* blooms at different stages of bloom formation and viral infection (12, 13, 15). Individual CTD casts were characterized and grouped into “early infection (EI),” “early infection revisited (EI_R_),” “late infection (LI),” or “post infection (PI),” using a combination of MODIS/AQUA satellite imagery, a suite of diagnostic lipid- and gene-based molecular biomarkers, analytical flow cytometry, *in situ* optical sensors, and sediment traps (12).

We further divided the “early infection” population into “early infection 1” and “early infection 2” in order to provide higher temporal sampling resolution for these parameters given the greater number of samples available at this site for analysis. CTD cast designations were thus as follows: EI_1_ (casts 50, 52, 56, 57, 63; 30 June – 3 July), EI_2_ (casts 68, 70, 72, 76; 4 July – 5 July), EI_R_ (casts 81, 84, 92, 93, 97; 7 July – 10 July), LI (casts 77 and 79; 6 July), and PI (casts 20, 25, 27, 33; 23 June 23 – 27 June). We also present data from three additional CTD casts not analyzed in the aforementioned study (12), along with an individual CTD cast (cast 92) from EI_R_ to illustrate a comparative signal for an early infected population. They include 29 (June 26), 40 (June 28), 89 (July 8).

Water was collected at 6 depths—extending from the subsurface, through the mixed layer encompassing the chlorophyll maximum, and down to 150 m—using Niskin bottles mounted on a 24-position rosette equipped with a Seabird SBE conductivity-temperature-depth (CTD) profiler. Sub-samples were stained with DAF-FM Diacetate, CM-H_2_DCFDA, and SYTOX Green (5 µM) as described above. Stained samples, along with an unstained control, were run on a Guava flow cytometer (EMD Millipore, Burlington, MA) in duplicate. We present data from 3 depths per cast corresponding to the depth at which *E. huxleyi* cell abundance was highest, along with one sampling depth above and one sampling depth below the *E. huxleyi* maximum, in box-and-whisker plots. These depths generally ranged from 8 – 40 m and are listed in Table S2.

*Data analysis and statistics*

Flow cytometry data collected for laboratory experiments were analyzed using FlowJo (v. 10.2). Statistics (counts and mean fluorescence) were based on at least 1000 *E. huxleyi* events. Mean fluorescence per cell for DAF-FM Diacetate and CM-H_2_DCFDA stained samples are reported as the difference between the mean fluorescence per cell of the stained sample and an unstained sample. Percent SYTOX Green positive cells are reported as the percent of the total *E. huxleyi* population that has elevated 520 nm fluorescence relative to an unstained control.

Flow cytometry data for fieldwork were analyzed using GuavaSoft InCyte (v. 2.2.2). *E. huxleyi* was distinguished by pre-gating all events by chlorophyll and gating the *E. huxleyi* population off side-scatter and forward scatter signatures corresponding to a reference culture. Statistics (counts and mean fluorescence) were based on at least 50 *E. huxleyi* events, with most samples encompassing 100-400 events, and averaged between two replicates per depth.

Statistically significant differences between infected and uninfected cultures for the parameters measured in this study were determined with Student’s t-tests (p<0.05). To test differences between multiple means, a one-way ANOVA with a Tukey HSD post-hoc test was used. Error bars on all graphs are + standard error of the mean (se). Linear regression analysis was used to explore relationships between various parameters in the NA-VICE dataset. All statistical tests were performed in R.

**2. Supplementary Figures & Tables**

**Figure S1.** Intracellular esterase activity of *E. huxleyi* CCMP1516 in EhV201 infected (dark grey bars) and control (light grey bars) over 72 h based on MUF-butyrate cleavage. Activity is expressed as the mean change in MUF fluorescence per min per µg protein (n=3, + se).


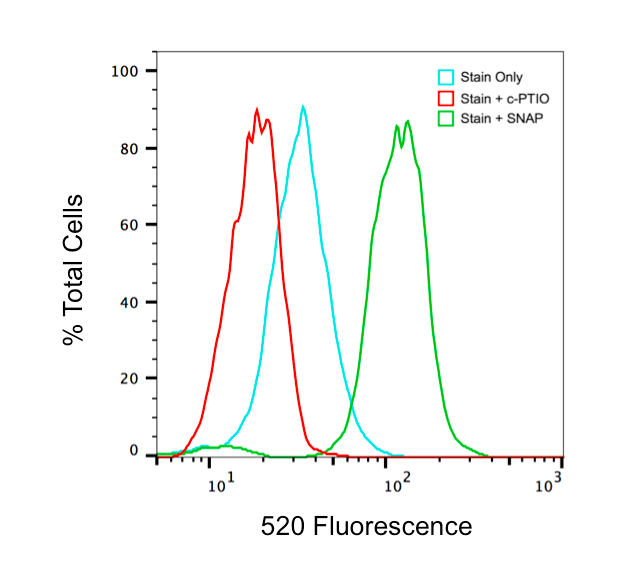


**Figure S2.** Histogram overlay of the 520 nm fluorescence of *E. huxleyi* CCMP1516 cultures stained with DAF-FM DA and either treated with the NO donor SNAP (250 µM, green), the NO scavenger c-PTIO (1 mM, red), or untreated (blue). Treatment with SNAP enhances DAF-FM DA fluorescence and c-PTIO diminished DAF-FM DA fluorescence.


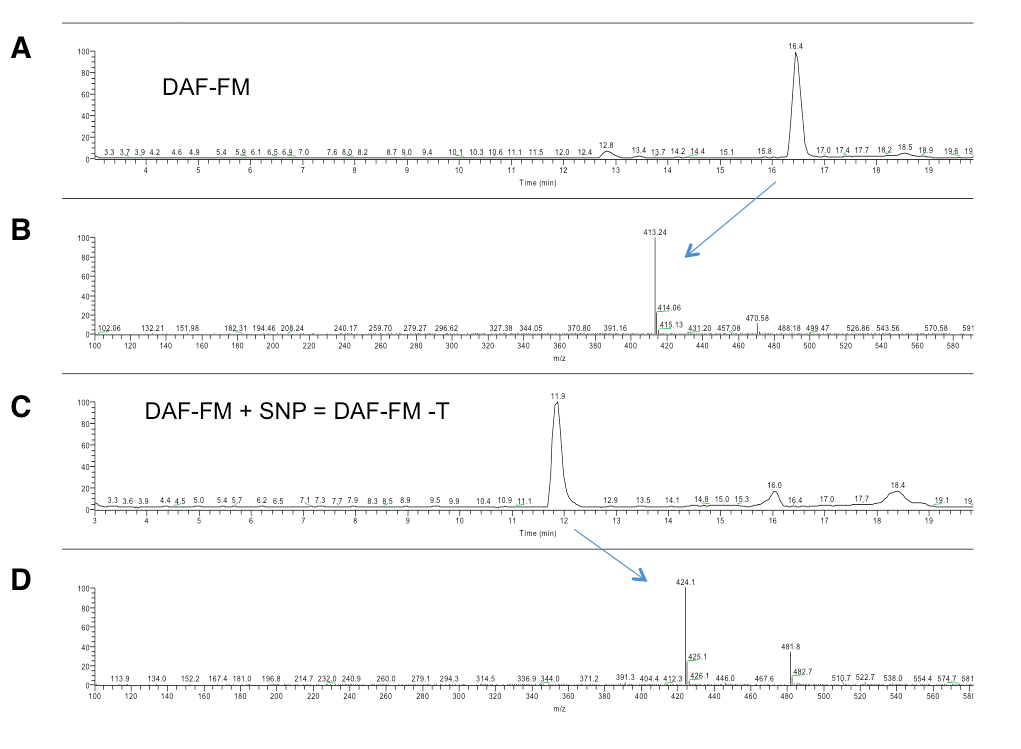


**Figure S3.** Full scan HPLC chromatograms showing peaks for DAF-FM (A) and DAF-FM-T (C), the product formed upon treatment of DAF-FM with the NO donor sodium nitroprusside (SNP).  Mass spectra of DAF-FM peak (B) and DAF-FM-T peaks (D) showing their diagnostic molecular ions.

**Figure S4.** Calibration curve of serial dilutions of DAF-FM-T standard (blue squares) detected and quantified by HPLC MS/MS overlayed by DAF-FM-T detected and quantified in *E. huxleyi* CCMP1516 cells treated with 100 µM of the NO donor SNAP (red circle) and 1 mM of the NO donor SNP (green triangle) for 1 h. Note that a 100 µM addition of SNAP to cells, a relatively high exogenous dose, lies close to the detection limit of DAF-FM-T.

**Figure S5.** Infection dynamics of *E. huxleyi* CCMP1516 cultures treated with various concentrations of the NO scavenger, c-PTIO. (A) Cell and (B) viral abundance of infected *E. huxleyi* cultures and an uninfected, non c-PTIO treated control (n=2, + se). Data are a representative subset of two separate experiments.

**
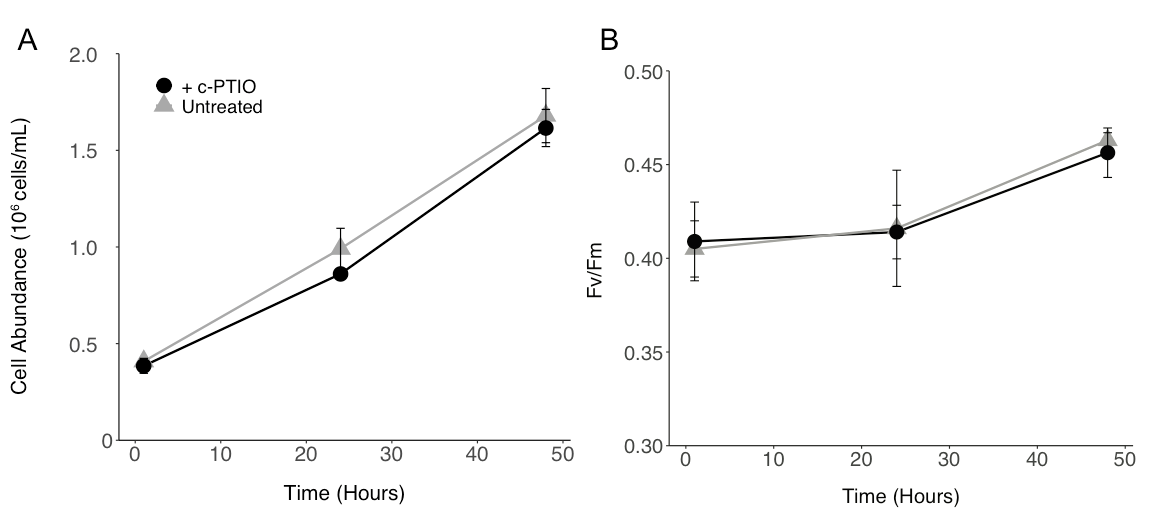
**

**Figure S6.** (A) Cell abundances and (B) F_v/_F_m_ values of uninfected *E. huxleyi* CCMP1516 cultures treated with c-PTIO (black, circles) or untreated (grey, triangles). Values are the mean of n=2 for untreated cultures and n=3 for c-PTIO treated cultures pooled from 2 replicates of 500 µM treatment and 1 replicate of a 1 mM treatment (+ se).

**Figure S7**: Determination of non-lethal doses of the NO donor, S-nitroso-N-acetylpenicillamine (SNAP), for exponentially growing *E. huxleyi* CCMP1516. (A) Cell abundance, (B) Fv/Fm, and (c) % SYTOX positive cells over 24 - 48 hours in cultures treated with 0, 1, 10, and 100 µM SNAP (n=3, + se).

**Figure S8**: DMSO only control of SNAP/H_2_O_2_ experiments. (A) Cell abundances and (B) F_v_/F_m_ of *E. huxleyi* CCMP1516 challenged with 100 µM H_2_O_2_, with and without pre-treatment with 0.1% DMSO, along with an untreated control (n=2, + se). Data are a representative subset of two separate experiments.

**Table S1**. Comparison of various physiological parameters of *E. huxleyi* CCMP1516 cultures incubated in the presence or absence of 25 µL of LEST for 3 h (n=1).


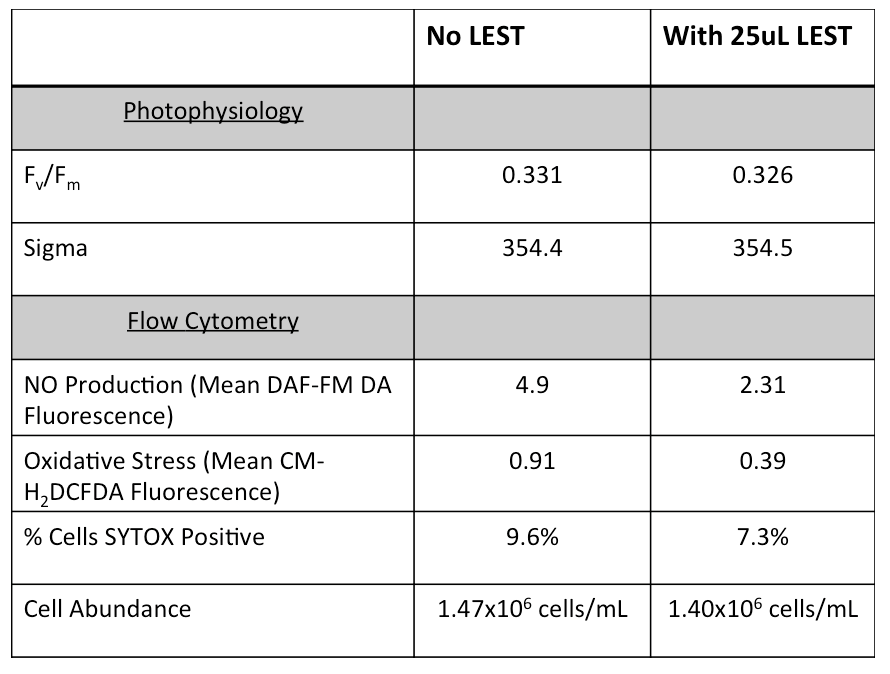


**Table S2.** Water depths sampled during NA-VICE that were used for the analyses presented in Figures 7 and Figure 8. Depths chosen at each station for detailed analysis correspond to the depth within the mixed layer that had the highest *E. huxleyi* cell abundance, along with one sampling depth immediately above and immediately below the E. huxleyi maximum. In cases where the highest *E. huxleyi* abundance was the first subsurface sample, the depths chosen were the subsurface and the next two depths sampled.

| **Cast #** | **Depths sampled (m)** |
| --- | --- |
| 20 | 8, 12, 16 |
| 25 | 8, 15, 25 |
| 27 | 5, 15, 26 |
| 29 | 5, 18, 28 |
| 33 | 5, 14, 21 |
| 40 | 11, 25, 34 |
| 50 | 9, 17, 25 |
| 52 | 4, 11, 21 |
| 56 | 8, 20, 31 |
| 57 | 11, 20, 30 |
| 63 | 17, 24, 31 |
| 68 | 10, 21, 30 |
| 70 | 9, 15, 22 |
| 72 | 20, 30, 40 |
| 76 | 6, 16, 20 |
| 77 | 8, 14, 20 |
| 79 | 7, 10, 20 |
| 81 | 5, 12, 20 |
| 84 | 7, 17, 24 |
| 89 | 7, 16, 25 |
| 92 | 14, 24, 34 |
| 93 | 9, 18, 25 |
| 97 | 8, 14, 22 |
